# Psychometric evaluation of the Lebanese Arabic version of the Dental Fear Survey: a cross-sectional study

**DOI:** 10.1186/s12903-021-02015-y

**Published:** 2021-12-18

**Authors:** Hiba Kassem El Hajj, Youssef Fares, Linda Abou-Abbas

**Affiliations:** Neuroscience Research Center, Faculty of Medical Sciences, Lebanese University, Beirut, Lebanon

**Keywords:** Dental Fear Survey, DFS, Psychometric properties, Lebanese, Arabic version

## Abstract

**Background:**

Dental fear is a prevalent problem that can lead to poor dental health. The Kleinknecht’s Dental Fear Survey (DFS) is one of the used scales to assess dental fear. The present study aims to evaluate the psychometric properties of the Lebanese Arabic version of the DFS (DFS-A) and to determine the optimal cut-off to identify dental fear as well as the correlates of dental fear in a group of Lebanese adults dental patients.

**Methods:**

A cross-sectional study was conducted among a group of 442 dental patients (18–65 years) recruited at 29 dental clinics from March to June 2019. Patients completed a questionnaire including questions about demographic characteristics, previous bad dental experience, trauma’s experience period, the sensation of nausea during dental treatment, the DFS-A scale, the Lebanese Arabic version of the Modified Dental Anxiety Scale (MDAS-A), and a general question about dental fear.

**Results:**

DFS-A revealed evidence of adequate psychometric properties. DFS-A scale demonstrated high internal consistency (cronbach’s alpha = 0.93). Test–retest reliability assessment demonstrated strong reproducibility of the DFS-A scale score (ICC = 0.92 with 95% CI (0.83–0.96), p value < 0.0001 (N = 30). Confirmatory factor analysis revealed a three-factor structure of the DFS-A reflecting fear associated with specific dental stimuli and procedures, patterns of dental avoidance and anticipatory anxiety, and physiologic arousal during dental treatment. A significant correlation was found between DFS-A and the MDAS-A indicating a good convergent validity. The optimal cut-off point to identify patients with and without dental fear is 41. Considering this cut-off score, the prevalence of dental fear in our sample was reported at 33.8%. Multivariable analysis showed that having previous scary and painful dental experiences, a sensation of nausea during treatment, and having dental anxiety were identified as predictors of dental fear**.**

**Conclusion:**

The adapted Arabic version of the DFS (DFS-A) is a valid tool to evaluate dental fear among Lebanese adult patients**.**

## Background

Dental fear is a prevalent condition that affects all populations worldwide [1]. It is defined as an “individual's response to an actual threatening event or a dangerous situation to protect one's life” [2]. Fear of dentistry and its expected results, avoidance of dental care, are problems of sufficient magnitude to cause concern among dentists [3, 4]. Before treatment, dentists should be able to detect the patient's level of fear so they can use appropriate management options.

To diagnose dental fear in clinical settings, it is important to have a valid and reliable diagnostic tool. In response to this need, researchers have developed various specific instruments to evaluate dental fear. One of the most used tool is the Kleinknecht’s Dental Fear Survey (DFS) [5]. DFS was introduced by Kleinknecht in 1973 containing 27 items [4], then it was reduced in 1984 to 20 items [5] reflecting avoidance of dental treatment, physiologic reactions to the dental treatment, fear aroused by different dental procedures. The DFS scale could act as a screening tool, among others screening tools, to identify these patients thus appropriate strategies could be applied to alleviate their fears such as some premedication (nitrous oxide, diazepam, antiemetic drug…), some relaxation techniques (muscle relaxation, diaphragmatic breathing…), appointments planning with less waiting time, distraction strategies such as watching movies during dental treatment and many others.

DFS was initially established in the English language and authors have revealed good psychometric properties supporting its validity and reliability among adults in the USA [4, 5]. Due to the influence of linguistic and cultural differences, DFS was translated and adapted to several languages including Indian [6], Greek [7], Japanese [4, 8, 9], Korean [10], Norwegian [11], Hungarian [12, 13], Brazilian [14, 15], Turkish [16], Chinese [17], and Malay [18]. The DFS was also translated and validated in the Arabic language by Alamri et al. and its psychometric properties were assessed in a sample of 12–15 year Saudi school students in Riyadh [19]. Results showed that the Saudi Arabian version of the DFS have high validity and reliability and confirm its feasibility to assess the dental patient’s anxiety and fear.

Cross cultural adaptation through testing Arabic translations of the measuring system in different countries can assign more information about its validity and reliability. So far, no studies have assessed the feasibility of the DFS in the cultural context of the Lebanese population. Thus, the current study aims to test the properties of the DFS Lebanese Arabic version and to identify the optimal cut-off to detect dental fear. In addition we sought to evaluate the factors associated with dental fear in a group of Lebanese adult dental patients.

## Methods

### Study design, setting, and population

A cross-sectional study was conducted over 4 months extending from March until June 2019 in different dental clinics from all Lebanese districts. Patients aged between 18 and 65 years who were able to read the Arabic language were included in our study. Pregnant women as well as patients with malignant diseases and mental disabilities were excluded. In addition, patients with missing information about DFS-A were excluded from the analysis (9 of 451 eligible patients). Thus, a total sample of 442 adult patients was collected. The study was approved by the Neuroscience Research committee at the medical Lebanese University (Reference number 12/2/2019). The guideline for Strengthening the Reporting of Observational Studies in Epidemiology (STROBE) was followed when reporting this study.

### Data collection

Information about the demographic characteristics (age, gender, marital status, educational level), previous bad dental experience, trauma’s experience period, perception of a periodontal problem, the sensation of nausea during dental treatment, the Arabic version of the Dental Fear Scale (DFS-A), the Arabic version of the Modified Dental Anxiety Scale (MDAS-A) were collected from the participants. To determine the test–retest reliability of the DFS-A scale, 30 patients were recruited to complete the DFS-A scale twice within 2 weeks. The methodology used in this study is described elsewhere. The questionnaires were distributed to 500 patients, 442 were completed which corresponded to an overall effective response rate of 88.4%.

### Study measurements

#### The Kleinknecht’s Dental Fear Survey (DFS)

This scale consists of 20 items divided into three components: Avoidance of dental treatment, physiologic reactions to the dental treatment, and fear aroused by different dental procedures [5]. Each question is provided with five possible answers ranging from 1 to 5. The Summation of answers constructed a score for the level of dental fear that ranges from 20 to 100. Avoidance of dentistry rated as follows (1 never to 5 often): Postpone making an appointment, canceled or failed to appear; felt autonomic arousal during dentistry rated as follows (1 none to 5 great): muscle tenseness, increased breathing rate, perspiration, nausea, heart beat faster; fear of situations and stimuli rated as follows (1 never to 5 very often): making an appointment, approaching the dental office, sitting in the waiting room, sitting in a dental chair, the smell of the dental office, seeing the dentist, seeing the anesthetic needle, feeling anesthetic needle, seeing drill, hearing drill, feeling drill, having teeth cleaned, and overall fear of dentistry. A DFS score of 53 represented the best compromise between sensitivity and specificity and was selected as the cut-off point for high dental fear [20].

The translation and Cross-cultural adaptation of the DFS-A, into the Arabic language, was performed according to the five steps proposed by Beaton et al. [21], after obtaining permission from the corresponding author. Two persons, whose native language is Arabic, first translated the DFS from English to Arabic. One translator was a professional sworn translator without any medical background and the other one was a dentist. Translators were asked to translate into a simple and comprehensible language for the Lebanese population. Discrepancies between the 2 translations were discussed to produce the Arabic version of the DFS. Another two professional sworn translators, who were blinded to the original English version, did back translations. Then, the research team reviewed the translated versions to develop a preliminary final version of the scale. This preliminary final version was administered to a sample of 15 Lebanese dental patients to test the meaning, comprehensibility, and acceptability of the scales’ items through individual interviews with these patients. On average, the questionnaires were completed within approximately 15 min. The meaning, comprehensibility, and acceptability of the scale’s items were discussed in individual interviews with the patients. After receiving the feedback from the patients, minor linguistic edits were made for one item to be more clear and comprehensible, a word “tool to drill” was added next to “the drill” in the DFS-A scale).

#### Modified Dental Anxiety Scale (MDAS)

The MDAS consists of 5 questions to measure the degree of dental anxiety in 5 situations: preparing for a dental visit, waiting in the dentist’s office for treatment, sitting in the dental chair for drilling, getting ready in the dental chair for scaling, and preparing for local anesthetic injection. Evaluations range from “non-anxious” [22] to “extremely anxious” [23]. The total score ranges from 5 to 25 while higher scores indicating severe anxiety. The Arabic version of the MDAS scale was validated among Arabic speaking populations in Saudi Arabia [25] and Lebanon [20].

### Statistical analysis

Data entry and analyses were performed using the statistical software SPSS version 21.0. Descriptive statistics were reported using means and standard deviations (SD) for continuous variables and frequency with percentages for categorical variables. Floor and ceiling effects were evaluated for the DFS-A total score. These effects were considered to be present if they exceeded 15–20% of patients receiving the minimum or maximum scores [24]. The Cronbach's alpha and composite reliability were calculated to assess internal consistency. A coefficient of above 0.7 indicated a good internal consistency. Test–retest reliability for the next occasion or appointment interval was evaluated using Spearman’s correlation on 30 patients. A correlation coefficient of 0.7 would act as a recommended threshold value [25]. Sample size guidance indicated that 5–10 participants per scale item would be adequate for establishing sufficient evidence of scale validity and reliability [26]. Thus, considering that the scale has 20 items, the required number of participants would be at least 100–200. In our study, the total sample size consisted of 442 participants. A post hoc power analysis was also computed to estimate the adequacy of the collected data (N = 442) to test the proposed logistic regression model. The power value was 0.99, which is greater than the recommended cutoff value of 0.80 [15]. Based on these a priori and post hoc analyses, our total sample size of 442 is appropriate for testing the proposed model”.

The total group was randomly divided into two groups using the randomization function on SPSS 22.0. In the first random-half sub-sample (n = 221), exploratory factor analysis was conducted using the principal components analysis with Varimax rotation. Sampling adequacy was assessed by the Kaiser–Meyer–Olkin (KMO) measure and Bartlett’s test of sphericity. The number of factors retained in the scale was determined based on Eigenvalues greater than 1, and visual inspection of the scree plot. Confirmatory factor analysis (CFA) was performed in the second random-half sub-sample (n = 221) using the Amos software version 22.0. The goodness-of-fit of the models were evaluated using Chi-square (χ^2^) and degrees of freedom (df), Root Mean Square Error of Approximation (RMSEA), and Comparative Fit Index (CFI).

Convergent validity using Spearman correlation was assessed to evaluate whether the total DFS-A scale was associated with MDAS-A. The discriminant validity of the scale was also assessed by comparing the means of the anxiety group and non-anxiety group using an independent-samples *t*-test. Clinical assessment of patients’ fear was obtained using a general question about dental fear as the gold standard. The optimal cut-off value for the DFS-A total score that can reliably differentiate dental patients with and without fear was determined using the receiver operating curve (ROC) analysis. The best cut-off point was identified by calculating the Youden index (Youden index = sensitivity + specificity − 1). The score at which the Youden index is maximal was considered the best cut-off point. The Pearson chi-square (*χ*^2^) test was used for statistical bivariate analysis. Multivariable logistic regression analyses were performed to identify associated factors of dental fear. Adjusted odds ratio and their 95% confidence intervals were reported. The final logistic regression model was reached after ensuring the adequacy of our data using the Hosmer and Lemeshow test. All statistical tests were two-sided, and the significance level was set at 0.05.

## Results

### Baseline characteristics of the study sample

Table 1 describes the sociodemographic characteristics of the study patients. The total number of patients was 442 of which 64.9% were females. Their mean age (SD) was 34.2 with an SD of 11.0. Of the total sample, 60.8% had university level of education. The mean of the DFS-total was 38.9 (SD = 17.4). The minimum and maximum total scores obtained from the DFS-A scale were 20 and 100 points. No significant floor and ceiling effects were detected as the proportion of patients reaching the minimum and maximum scores were 9.3% and 0.2% respectively.

**Table 1:** Socio-demographic characteristics of the study participants (N = 442)

	All sample (N = 442)
Age mean (SD)	34.2 (11.0)
*Gender n (%)*	
Male	155 (35.1)
Female	287 (64.9)
*Educational level n (%)*	
Primary	26 (5.9)
Complementary	75 (17.0)
Secondary	72 (16.3)
University	269 (60.8)

N or n Frequency, % percentage, *SD* Standard deviation

### Reliability of the DFS-A

The internal consistency of the DFS-A was assessed using Cronbach’s alpha coefficients and composite reliability (CR) coefficients. The DFS-A scale showed high internal consistency with a Cronbach's alpha = 0.93 and a composite reliability of 0.94. The Corrected–item to total correlation coefficients varied from 0.45 to 0.82. Removing an item from the construct did not significantly affect the Cronbach's alpha which ranged between 0.93 and 0.94 (Table 2). Spearman's rank correlation coefficient was used to assess test–retest reliability or score consistency over time for 30 patients. The correlation coefficient for the total DFS-A score was 0.7 with a p-value < 0.0001 suggesting an acceptable reproducibility over time.

**Table 2:** Internal consistency of the DFS-A (N = 442)

Item-total statistics	Scale mean if item deleted	Scale variance if item deleted	Corrected item-total correlation	Cronbach's alpha if item deleted
1. Postpone making an appointment	37.25	293.88	0.69	0.93
2.Canceled or failed to appear	37.57	302.22	0.61	0.93
3.Muscle tenseness	36.99	299.04000	0.49	0.93
4.Increased breathing rate	37.33	297.62	0.62	0.93
5.Perspiration	37.55	303.57	0.55	0.93
6.Nausea	37.46	304.6	0.48	0.93
7. Heart beat faster	37.21	293.73	0.65	0.93
8.Making an appointment	37.17	273.52	0.45	0.94
9.Approaching dental office	37.4	293.29	0.78	0.93
10.Sitting in the waiting room	37.04	289.28	0.58	0.93
11.Sitting in dental chair	36.91	285.27	0.82	0.92
12.Smell of dental office	37.27	299.01	0.58	0.93
13.Seeing the dentist	37.47	298.40	0.67	0.93
14.Seeing anesthetic needle	36.49	287.18	0.70	0.93
15. Feeling anesthetic needle	36.35	288.40	0.67	0.93
16.Seeing drill	36.59	283.45	0.77	0.93
17.Hearing drill	36.51	284.47	0.75	0.93
18. Feeling drill	36.5	285.13	0.71	0.93
19.Having teeth cleaned	37.37	299.3	0.64	0.93
20.Overall fear of dentistry	36.94	287.40	0.81	0.93

### Construct validity of the DFS-A Scale

The exploratory factor analysis of the DFS-A scale showed a KMO measure of 0.92 indicating adequate sampling adequacy and a highly significant Bartlett’s Test of sphericity (χ^2^ = 3205.10, df = 190, p value < 0.0001). The scree plot of the Eigen values revealed a three-factor structure of the DFS-A scale: the first factor was related to fear of specific stimuli/procedures (item 14–20) and accounted for 25.56% of the scale’s total variance, the second factor was associated with patterns of dental avoidance and anticipatory anxiety and accounted for 22.65% of the scale’s total variance (items 1, 2, and 8–13), and the third factor concerned physiological arousal during dental treatment and accounted for 16.22% of the scale’s total variance (items 3–7). The factor loadings for each item are presented in Table 3. An exploratory factor analysis using parallel bootstrapping to derive simulated eigenvalues from random samples for comparing with the observed data was also conducted. Random eigenvalues derived from the bootstrapping procedure showed that 2 factors would have been selected (eigenvalues of 1.57 and 1.47).

**Table 3:** Exploratory factor analysis of the DFS-A Scale

Items	Factor 1	Factor 2	Factor 3	Communality
Overall fear of dentistry	0.634			0.749
Seeing anesthetic needle	0.771			0..675
Feeling anesthetic needle	0.781			0.659
Feeling drill	0.783			0.761
Seeing drill	0.840			0.815
Hearing drill	0.840			0.800
Having teeth cleaned	0.595			0.547
Smell of dental office		0.644		0.611
Postpone making an appointment		0.640		0.641
Sitting in the waiting room		0.627		0.605
The smell of the dentist’s office		0.510		0.413
Sitting in the dental chair		0.607		0.767
Making an appointment		0.566		0.400
Seeing the dentist walk in		0.644		0.589
Approaching dental office		0.761		0.775
Muscle tenseness			0.654	0.611
Nausea			0.616	0.527
Perspiration			0.780	0.645
Heartbeat faster			0.703	0.663
Increased breathing rate			0.736	0.699
Eigenvalue	9.90	1.85	1.13	
Percentage of explained variance	25.56	22.65	16.23	

**Fig. 1:**
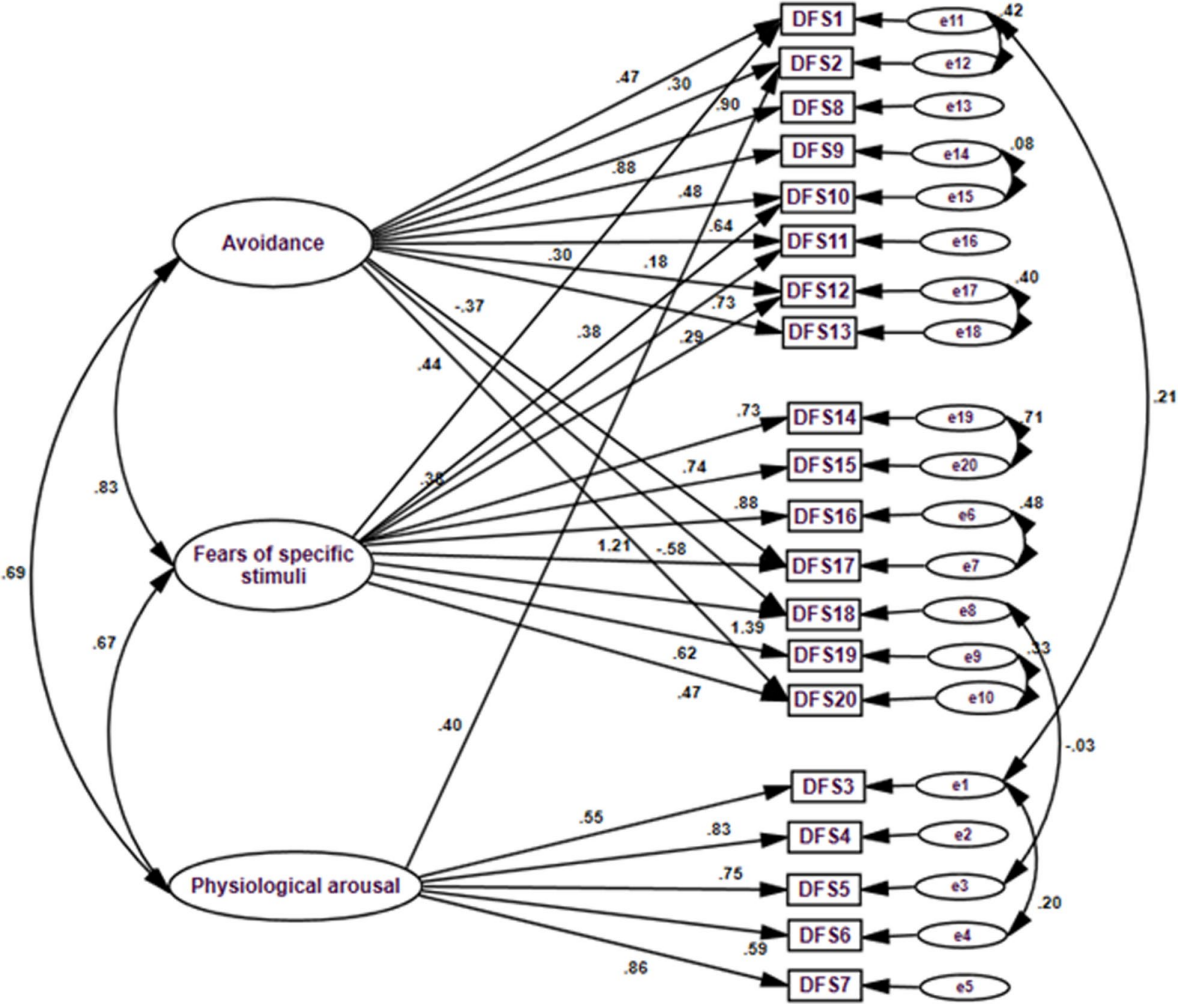
Three-factor model of the Arabic version of the Dental Fear Survey (DFS-A)

A Confirmatory factor analysis was performed to determine the multidimensionality model of the DFS-A. A priori hypothesized model, that is the 20 items of the instrument load in two factors as suggested by the EFA, did not fit the data well. Inspection of the three-factor model also displayed an unsatisfactory fit. The goodness of fit statistics were NFI = 0.793, CFI = 0.826, and RMSEA = 0.134. Inspection of the modification indices suggested adding error covariance between DFS1 and DFS2, DFS1 and DFS3, DFS3 and DFS6, DFS6 and DFS15, DFS9 and DFS10, DFS 12 and DFS 13, DFS 14 and DFS 15, DFS 16 and DFS 17, DFS19 and DFS20. In addition, Paths from Factor I to DFS 17, DFS 18, and DFS 20 were added. Paths from Factor II to DFS1, DFS10, DFS11, and DFS12 and a path from Factor III to DFS 2 were also added. These modification resulted in a significant improvement of the fit indices. The goodness of fit statistics were NFI = 0.923, CFI = 0.958, and RMSEA = 0.06. All standardized factor loadings for the one-factor model were significant at p < 0.01 (Fig. 1).

### Convergent validity of the DFS-A

Table 4 displays the correlation between the Modified Dental Anxiety Scale (MDAS-A) and DFS-A total scale. Statistically significant correlations were found between MDAS-A total scale and DFS-A (r = 0.8, p value < 0.0001), indicating that the DFS-A has good convergent validity.

**Table 4:** Spearman correlation between MDAS-A and DFS-A

Measure	Spearman coefficient	P value
DFS-A total score	0.8	< 0.0001
Anticipating a visit to a dental clinic	0.84	< 0.0001
Waiting in the dentist's office for treatment	0.84	< 0.0001
Waiting in the dental chair for drilling of teeth	0.89	< 0.0001
Waiting in the dental chair for scaling the teeth	0.77	< 0.0001
Waiting in the dental chair for receiving a local anesthetic injection	0.85	< 0.0001

*MDAS-A* The Arabic version of the Modified Dental Anxiety Scale, *DFS-A* The Arabic version of the Dental Fear Scale; P-value < 0.05 is considered significant

### Criterion validity of the DFS-A

**Fig. 2:**
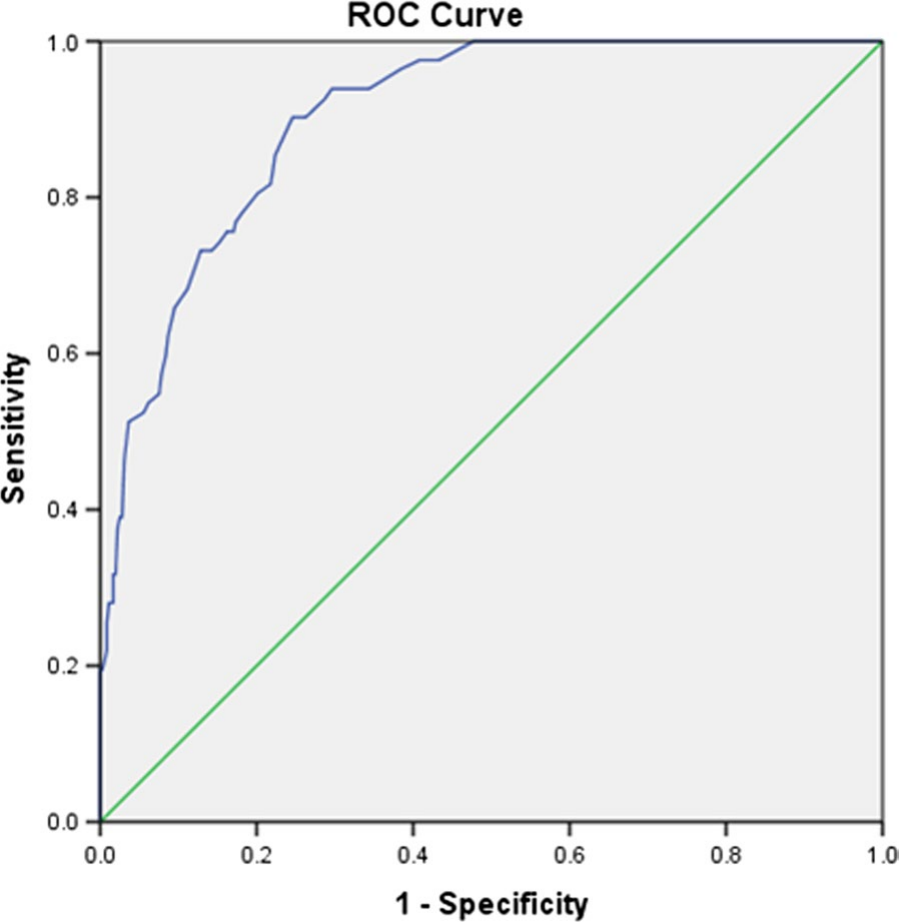
Receiver operating curve (ROC) curve showing sensitivity as a function of 1-Specificity of the Dental Fear Survey (DFS-A)h

Mean scores on the DFS-A scale were compared between those diagnosed with and without fear (made through the general question about dental fear diagnosis) using the Mann–Whitney test. A statistically significant mean rank difference was found between the two groups with higher scores for fearful compared to the non-fearful group (P value ˂ 0.0001) indicating that DFS-A has good discriminant validity. The receiver operating curve (ROC) analysis was also conducted to evaluate the diagnostic validity of the DFS-A scale. The area under the curve (AUC) was 0.9 (95% confidence intervals 0.87–0.93) indicating a good diagnostic accuracy of the DFS-A (Fig. 2). A cut-off score of > 41 was identified as the best score to distinguish between patients with and without fear with a sensitivity of 90 and a specificity of 75.

### Assessment of dental fear and its associated factors

Of the total, 33.8% of the participants have reported having dental fear. Overall, previous scary and painful dental experience, a sensation of nausea during dental treatment, and dental anxiety were identified as predictors of dental fear (Table 5).

**Table 5:** Factors associated with dental fear among Lebanese dental patients

	All (N = 442)		
	OR	95% CI	p value
*Dental experience*			0.007*
No experience	1.00		
Scary	3.69	1.07–12.71	
Embarrassing	0.86	0.14–5.49	
Painful	3.62	1.64–7.99	
Wrong treatment	0.95	0.41–2.21	
*Sensation of nausea during dental treatment*			< 0.0001*
No	1.00		
Yes	4.52	2.23–9.15	
*Having dental anxiety (MDAS score* > *10)*			< 0.0001*
No	1.00		
Yes	18.71	10.07–34.77	

Factors entered into the model: age, gender, marital status, educational level, previous bad dental experience, the trauma’s experience period, the perception of a periodontal problem, the dental anxiety as reported by MDAS-A and the sensation of nausea, *P value < 0.05 is considered statistically significant

N frequency, *ORadj* adjusted odds ratio, *CI* confidence interval, *MDAS* Modified dental Anxiety scale

## Discussion

The present study was conducted to evaluate the psychometric properties of the Lebanese Arabic version of the DFS and to identify the factors associated with dental fear. Results showed evidence of good validity and reliability of the scale that can be used to assess dental fear in Arab-speaking individuals. Having previous scary and painful dental experiences, a sensation of nausea during dental treatment, and having dental anxiety were identified as predictors of dental fear**.**

The results showed an adequate level of internal consistency with a Cronbach’s alpha of 0.93 for DFS-A, indicating the homogeneity of the scale items. The internal consistency of the translated versions of the DFS was 0.95 for the Brazilian version [15], ranged from 0.94 to 0.96 for the Japanese version [8], 0.94 for the Chinese version [17] and 0.96 for the Greek version [7], which were consistent with our results.

Test–retest reliability of the DFS-A, as assessed by the ICC (0.92) was high reflecting stability over time. This is also concordant with the results of the Oliviera study where the ICC results were 0.882 (95% CI 0.793–0.930) for avoidance, 0.874 (95% CI 0.810–0.917) for physiological arousal, and 0.897 (95% CI 0.829–0.937) for fears of specific stimuli/situations [15]. This value was 0.85 for the Chinese version [17], 0.97 in a Norwegian study [11].

The exploratory factor analysis revealed three factor structure of the DFS-A three constructs underlying dental fear namely “fear of specific stimuli/procedures”, “patterns of dental avoidance and anticipatory anxiety” and “physiological arousal during dental treatment” together explaining about 64% of the variance. Studies that inspected the factor structure of the DFS have shown inconclusive results, with some showing best fit for a three-factor structure [4, 7, 8, 15, 27], one showing best fit for a four-factor structure [17] and another one for a six-factor structure [28]. In our study, the cross-cultural validity of the DFS-A was established by testing the hypothesis that the structure of the translated instrument was in concordance with the original U.S. version. Our results were consistent with the results of the original English version of the DFS revealing a three-factor structure [5]. Nevertheless, a more sophisticated model that takes into consideration logical correlations between items and the latent variables demonstrated an improvement in fit statistics compared with the hypothetical model. Similar approaches were adopted in the studies conducted in Japan and Brazil in which different error covariances and paths were added to improve the model fit. However, Sirin et al. considered a new distribution pattern of 18 items only instead of 20 items, which resulted in a better statistical fit [29]. Given the aforementioned, the DFS factor structure is still questionable and may potentially vary due to the cultural differences between the populations. This indicates a need for further research in large sample size to confirm the DFS factor structures in various samples. Until reaching conclusive results on the optimal factor structure for the DFS-A, researchers would be wise to conduct their own factor analysis in order to determine whether the three dimensions representing “fear of specific stimuli/procedures”, “patterns of dental avoidance and anticipatory anxiety” and “physiological arousal during dental treatment are applicable for their sample.

The discriminant validity of the DFS-A scale was confirmed using the ROC curve. The cut-off score of > 41 was identified as the best score to differentiate between patients with and without dental fear with a sensitivity of 90% and a specificity of 76%. The Brazilian DFS reported a different cut-off of 53 that met a sensitivity of 88.9% and a specificity of 92.5% [20]. This could be explained by the cultural difference between populations, hence the importance to identify the optimal cut-off that is able to discriminate patients with and without dental anxiety among different populations.

Having previous scary and painful dental experiences, reporting having a sensation of nausea during dental treatment, and having dental anxiety were found to be risk factors for having dental fear. The previous scary and bad dental experiences can not be erased from the memories of a young brain, so attention should be made when treating young patients, since this will influence their behavior in all the upcoming dental appointments. Usually pedodontic dental practitioners are trained to deal with young dental patients and especially difficult patients by prescribing some antihistaminic drugs that alleviate the patient, using nitrous oxide, benzodiazepines and sometimes treating the patient under general anesthesia. Furthermore, nauseated patients have been proved to suffer from dental fear. This could be related to the difficulty in accepting some dental treatments, in the worst cases, all dental treatments, taking intra-oral radiographs and sometimes brushing their teeth. This disturbance while treating their teeth could lead to dental fear and consequently avoiding dental treatment which will worsen their dental health. Anti-emetic drugs can be prescribed to alleviate their nausea, one hour before the appointment. Finally, being anxious about dental treatment can lead to being afraid of dental treatment. In the literature, these two expressions were used interchangeably [30] although anxiety is towards an unknown and a new dental experience. Yet fear is toward a previous unpleasant dental experience. This anxiety towards an unknown future dental experience can lead to being afraid of this dental experience, based maybe on previous bad dental experience or dental attitudes, and sometimes it will result in avoiding dental treatment. It's worth mentioning that women in our sample expressed more dental fear than men (data not shown). However, gender differences in dental fear disappeared once we controlled for confounder variables, suggesting that having previous scary and painful dental experiences, reporting having a sensation of nausea during dental treatment, and having dental anxiety fear of the COVID-19 pandemic, rather than gender per se, drives dental fear differences. A plausible explanation for our findings could be related to Lebanese cultural norms or the higher response rate reported by females compared to males, both of which could have had a significant impact on the study's results. Thus, additional studies with larger sample size are needed to draw robust conclusions.

Given that dental fear is an international problem that may lead to avoiding dental treatment, it is important to have a valid and reliable scale to identify patients with dental fear. The DFS-A scale could act as a screening tool to identify these patients thus appropriate strategies could be applied to alleviate their fears. Several methodological limitations can impact the results of this study such as the possibility of selection bias due to the convenience sampling strategy that was applied to recruit patients. It is worth noting that this translated Arabic-language form may not be suitable for other Arabic-speaking communities. The linguistic characteristics of other Arabic-speaking societies may impose some adjustments to the scale.

## Conclusion

This study was the first to explore the psychometric properties of the Arabic version of the DFS in Lebanon. Results revealed that the DFS-A has good validity and reliability. Therefore, it is considered a useful screening way for assessing dental fear among Lebanese adult patients in clinical settings. For this population, having previous bad scary and painful dental experience, reporting having a sensation of nausea during dental treatment, and diagnosed as having dental anxiety are risk factors for having dental fear.

## Data Availability

Data are available from the corresponding authors upon reasonable request.

## Abbreviations

DFS: The Kleinknecht’s dental fear scale
DFS-A: Lebanese Arabic version of the dental fear scale
MDAS-A: Lebanese Arabic version of the Modified Dental Anxiety Scale
SPSS: Statistical Package for Social Sciences
SD: Standard deviations
EFA: The exploratory factor analysis
CFA: Confirmatory factor analysis
χ2: Chi-square
df: Degrees of freedom
RMSEA: Root mean square error of approximation
GFI: Goodness of Fit Index
CFI: Comparative Fit Index
ROC: The receiver-operating curve
ICC: Intra-class correlation coefficient
KMO: Kaiser–Meyer–Olkin test
EFA: Exploratory factor analysis
AUC: Area under the curve
OR: Odds ratio
CI: Confidence interval
